# Aquaponics production system: A review of historical perspective, opportunities, and challenges of its adoption

**DOI:** 10.1002/fsn3.3154

**Published:** 2022-12-18

**Authors:** Victor Tosin Okomoda, Sunday Abraham Oladimeji, Shola Gabriel Solomon, Samuel Olabode Olufeagba, Samuel Ijabo Ogah, Mhd Ikhwanuddin

**Affiliations:** ^1^ Department of Fisheries and Aquaculture, College of Forestry and Fisheries Joseph Sarwuan Tarka University (Formerly, Federal University of Agriculture Makurdi) Makurdi Nigeria; ^2^ Higher Institution Centre of Excellence, Institute of Tropical Aquaculture and Fisheries Research (AQUATROP) Universiti Malaysia Terengganu Kuala Nerus Terengganu Malaysia; ^3^ Agricultural Department National Biotechnology Development Agency (NABDA) Abuja Nigeria; ^4^ Department of Aquaculture, Faculty of Agriculture Federal University Gashua Gashua Nigeria

**Keywords:** green farming alternative, hydroponics, recirculatory system, vegetable

## Abstract

The aquaponics production system integrates hydroponics and recirculatory aquaculture system for the simultaneous production of plants and fish. At a time, such as the postpandemic era, the aquaponics system represents an efficient green farming and eco‐friendly alternative to sustainable agricultural production. In this review, the history and development of the production systems were traced vis‐a‐vis its pros and cons. Although there has been much dispute about the origin of the system, the numerous records of developmental attempts in history have all led to the current complexity of the systems and their efficiency. Water conservation, improved performance, food security, less pollution, and low energy consumption are some of the advantages identified in the use of aquaponics systems for food production. Challenges to the domestication of the system, however, include moderately high start‐up capital, the need for stable electricity to operate the system, nutrient availability, as well as treatment of diseases in the system. Although the aquaponics production system could be a panacea for food security in Africa, modalities for the domestication of this technology are largely not in place, hence the need for some government interventions in this regard.

## Graphical abstract

Aquaponics Production system is a green alternative and ecofriendly food production system. Domestication of this tech in third‐world countries would need serious Government intervention.

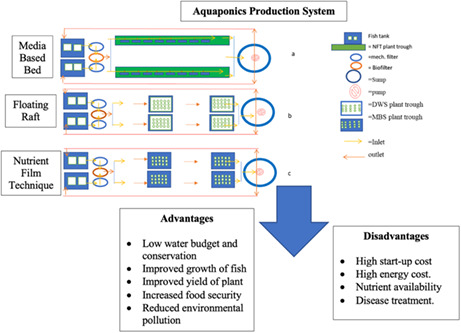

## 1 INTRODUCTION

Aquaculture being the fastest‐growing food‐producing sector in the world employs different kinds of production systems (FAO, 2019). Among these systems, aquaponics is considered one of the most efficient and environmentally sustainable methods of the 21st century (FAO, 2014; Oladimeji, Olufeagba, et al., 2020; Oladimeji, Okomoda, et al., 2020); it is simply the combined culture of fish and plants in a recirculating system. Mainly, recirculatory aquaculture systems are designed to raise large quantities of fish in relatively small volumes of water by treating the water to remove toxic waste products and reuse again for fish culture (Rakocy & Hargreaves, 1993; Timmons et al., 2010; Verdegem, 2013). Although the nitrifying bacteria convert toxic ammonia and nitrite into less toxic nitrate in specialized filters called biofilters, however, if there are disturbances to the biofilter or ammonia production that exceeds the capacity for the biofilter, they can accumulate to levels that are deleterious to the fish (Rakocy & Hargreaves, 1993; Yildiz et al., 2017). Moreover, nitrifying bacteria are incapable of removing nitrate, thus leading to a gradual accumulation of this compound to levels that may require water exchange to mitigate their negative effects (Konschel, 2009). This is where the integration with hydroponics become important in removing the organic matter for use by plants and purifying the water for reuse by fish.

The integrated technique of the aquaponics system is therefore perceived to be a more efficient agricultural technology than the individual systems (McGuire & Popken, 2015; Silva et al., 2017). The use of an aquaponics system not only removes nitrate from the system but also converts otherwise toxic nitrogenous waste into forms usable by plants which are sold to get additional income for the farmer (Pantanella et al., 2010). Consequently, this has the potential to use less water, which can be a limiting resource in some areas. Also, the hydroponic component of the aquaponics system over time is populated with appropriate microbiota which helps in biofiltration. Therefore, this improves the efficiency of water purification beyond what a separate biofilter in conventional recirculating systems could achieve (Rakocy & Hargreaves, 1993). Consequently, the aquaponics system has several edges over the conventional recirculating aquaculture and agriculture systems.

However, balancing the nutrients obtained from the fish system with the requirements of the hydroponic plants is key to optimizing the utilization of resources and productivity of the system (Rakocy et al., 2006a, 2006b). This has been demonstrated in many studies with objectives to improve the functionality of the system (Castro et al., 2006; Oladimeji, Olufeagba, et al., 2020; Oladimeji, Okomoda, et al., 2020). The ratio of fish‐to‐plant production has been calculated by Endut et al. (2010) to be 15–42 g of fish feed/m^2^ of plant‐growing area. With this, the nutrient generated from fish and the nutrient removed by the plants are perfectly balanced. Above or below this optimum ratio, the plant performance is significantly affected negatively. Aside from the improved functionality, the feasibility of getting financial incentives through the aquaponics production of fish and crops has been well documented (Rakocy et al., 2004). Aquaponic production has been reported to produce six times more yields on one‐sixth of space and only require one‐sixth of water for production compared to conventional outdoor agriculture and aquaculture facility (Nelson & Pade, 2017).

Selecting suitable plant species and fish for aquaponics systems, however, is based on various parameters which include but are not limited to the tolerance ability of both plant and fish to high nutrient levels, availability of space, as well as the possibility of all year‐round production of the target commodity (Enduta et al., 2011; Patil et al., 2016; Rakocy et al., 2004). Some of the food crops tested for their suitability for culture in the aquaponics system using fish effluent as their primary fertilizer are shown in Table 1 below. Sadly, not much has been done with indigenous African crops as regards rearing in the aquaponics system. This may be justified by the fact that the adoption and development of the system in the continent is still in its infancy.

**TABLE 1:** Some aquaponic crops and fish in previous studies

Fish(es) used	Plant(s) used	Findings	References
*Oreochromis* sp.	*Ipomoea aquatic*	Increased feeding frequency favored stable and good water quality and fastened fish growth and plant growth by as much as 4.9% and 11%, respectively	Liang and Chien (2013)
*Oncorhynchus mykiss*	*Coriandrum sativum* L., *Petroselinum crispum, Lactuca sativa*, and *Plantago coronopus*	Comparative performance overview of stand establishment expected harvest biomass and time to harvest for the various pisciponic crops	Buzby et al. (2016)
*Oreochromis sp, Clarias gariepinus*	*Ocimum basilicum, Origanum majorana*, and *Petroselinum crispum*	Effects of fish species on plant growth and vice versa	Knaus and Palm (2017)
*Oreochromis niloticus* × *Oreochromis mossambicus*	*Ipomea aquatic*	Membrane filtration treatment was shown to have a positive effect on the performance of recirculating pisciponic system	Wang et al. (2016)
*Clarias gariepinus*	*Amaranthus* spp. and *Ipomea aquatic*	Optimization of the pisciponics system for selected fish and plant species	Mamat et al. (2016)
*Osphronemus goramy*	*Lactuca sativa* L. var. *longifolia*	Gouramy waste could be processed by phytoremediation for growing romaine lettuce in a pisciponic system	Purwandari et al. (2017)
Huso × *Acipenser ruthenus*	*Lactuca sativa*	Optimization of the pisciponics system for selected fish and plant species	Dediu et al. (2012)
*Clarias gariepinus*	Pumpkin *Telfairia occidentalis*	Comparative study favors aquaponics production of catfish and pumpkin over conventional farming methods	Oladimeji, Olufeagba, et al. (2020)
*Clarias gariepinus*	Pumpkin *Telfairia occidentalis*	Optimization of the growth media for the effective production of catfish and pumpkin was done. Agricultural by‐products serve perfectly as growth media	Oladimeji, Okomoda, et al. (2020)
Lemon fin barb hybrid (*Hypsibarbus wetmorei ♂ × Barbonymus gonionotus ♀*)	Chinese celery (*Apium graveolens*), coriander (*Coriandrum sativum*), and peppermint (Mentha × piperita)	Biological filtration of some herbs shows that peppermint was superior in terms of gross biomass and water purification compared to others	Ogah, Kamarudin, Nurul Amin, and Puteri Edaroyati (2020)
Lemon fin barb hybrid (*Hypsibarbus wetmorei ♂ × Barbonymus gonionotus ♀*)	Peppermint (Mentha × piperita)	Additional night lighting favors the production characteristics of peppermints	Ogah, Kamarudin, Nurul‐Amin, and Edaroyati (2020)

The development and wide adoption of the system in Africa is key to solving some of the problem faced on the continent. Beyond the system's ability to improve food security through the production of diversified food commodities, the aquaponics system also presents a unique solution to the age‐long deadly resource‐use conflict between farmers and herdsmen in most parts of Africa (Ajuwon, 2004; Fasona & Omojola, 2005; Udo et al., 2019). This is because the production of fish and crops can be done within a small space with high efficiency. Although ranching has been suggested as part of the solution to the farmers–herders’ crisis, this in itself presents a unique challenge of sourcing, storing, and maintaining quality fodder for the optimum growth of large numbers of cattle restricted in different locations. Aquaponic also could be used to produce quality cattle fodder, however, no one has reported the production of fodder in the aquaponic system to date.

## 2 HISTORICAL PERSPECTIVE OF THE AQUAPONICS PRODUCTION SYSTEM

There has been much dispute about the origin story of the aquaponics system; however, many records trace the pilot forms of the systems back to the days of the medieval Aztecs inhabiting inner Mexico in 1000 AD (Shabeer, 2016). These Aztecs were said to have developed the first version of the aquaponics production system because they did not have sufficient land to grow their food. In their “archaic” approach to solving this land problem, they constructed rafts that were covered with soil to enable the planting of vegetable crops. These were termed “floating farms” and represented the earliest forms of aquaponics systems designed to produce food (Jones, 2002). However, up till this point, the production system seemed to be descriptive of a simple soil‐less culture rather than an aquaponic system. The introduction of fish into the established system described above could be linked to farmers in South China and Thailand who cultured suitable fish species alongside rice in paddy fields (Shabeer, 2016). Another variant would be the Chinese farmers rearing ducks in cages located above rearing tanks of fin fishes. Hence, the duck's fecal droppings were used to feed the fish, while the feces of the fish and wastewater were transferred into a catfish tank and subsequently into the rice crops (Rakocy et al., 2004).

In 1969, William McLarney, Nancy, and John Todd built a prototype replica of the Aztec's aquaponic system (with some modifications) to provide shelter, vegetables, and fish throughout the year (Shabeer, 2016). The numerous records of developmental attempts in the history of aquaponic production systems all have led to the current level of efficiency and systems prototype we have today. Experts at the New Alchemy Institute and the North Carolina State University are reputed to be behind modern aquaponics. Motivated by the quest to reduce overdependency on finite natural resources such as land and water, these scientists developed the modern and efficient integration of the aquaculture and hydroponics system. Most of the research on aquaponics production systems began in the early 70s with scientists setting the pace for research that we follow even to this day. The probably most popular commercial‐scale aquaponics set up in the 1980s was by Dr. James Rakocy and his team at the University of the Virgin Islands (UVI). The survey conducted by Love et al. (2014) shows that the aquaponics production system's popularity has grown since the 80s, justifying its increasing significance as regards improving food security using innovative approaches.

Recent advances in aquaponic research have opened additional possibilities, with stakeholders developing brilliant and diverse aquaponics operational models for viable and practical food production (Graber & Junge, 2009; La Crosse et al., 2017). This includes the development of deep‐water culture hydroponics and the adoption of biogas as an energy source for the system. All of these showcase the excellent creative ideas by aquaculture stakeholders in keeping alive the dream of expanding and revolutionizing aquaponics production systems. With the progressive need to feed the ever‐growing human population (Chan et al., 2017; FAO, 2016; Junge et al., 2017; Mancuso, 2014), it is hoped that aquaponics production will play a big role in cutting short the wide gap in the demand for fish. It is noteworthy also that the consumption of vegetables tends to increase as average human wages increase (Smith, 2010). More so, the increasing awareness of the health benefit of eating vegetables and cutting down on red meat connotes that the per capita vegetable and fish consumption in many countries is projected to increase in the next few decades (FAO, 2019).

To accommodate this fast‐growing demand for higher‐quality food products (i.e., fish and vegetables), technological advances in aquaponics will become increasingly necessary. This presents greater opportunities for the farmers to target this broadening market of the future, particularly in urban areas where land is scarce but, on the other hand, population densities are high (McGuire & Popken, 2015). In Nigeria, not much has been done in terms of research and popularization of the aquaponics production system despite its advantages. Only recently was the production of Pumpkin *Telfairia occidentalis* reported using different waste materials as grow beds and cultured with African catfish *Clarias gariepinus* (Burchell, 1822) (Oladimeji, Olufeagba, et al., 2020). Hence, aquaponics production research in Nigeria and many parts of Africa is still underdeveloped. There is a need to test the suitability of many African vegetables in production with popularly cultured fish species in the country.

### 2.1 Types of aquaponics systems

A typical aquaponics system consists of fish tanks for the aquaculture component; the grow bed or trough for the hydroponic culture, and the filtration arm for bio‐ and mechanical filtration (Love, Fry, et al., 2015). Generally, the different model of the aquaponics system is determined by the types of hydroponic unit used. These are classified into three types, namely media‐based bed, floating raft, and nutrient film technique. Among them, the media‐based system is believed to be more efficient in the utilization of nitrogen since it provides more volume‐to‐surface area ratio for the microbes than the other two types (Lennard & Leonard, 2006). In an international survey by Love et al. (2014), 86% of the respondents adopted media‐based planting units because they considered it to be the most common and popular method for raising crops. The strength of the nutrient film technique system over others is its better ability to provide higher oxygen to the plant roots, hence facilitating a high yield of vegetables (Liang & Chien, 2013).

However, the nutrient film technique has been critiqued as being only suitable for small vegetable species because it cannot support plants with high quantities of roots due to their potential to blockage the recirculation flow (Cherif et al., 1997; Engle, 2015). Effective removal of solid waste is a critical factor in a nutrient film system to prevent the clogging of the growing bed channel. This is a major challenge that is naturally overcome by using the floating‐raft‐type system. Also, the nutrient film technique and floating‐raft aquaponics systems require a biofilter as well as a sedimentation tank for the nitrification and removal of solid waste, respectively (Nelson, 2008). The media‐filled type, however, is the simplest system which does not necessarily require separate biofilters because it contains substrate or media (e.g., pumice stones or clay beads) in the grow bed which serves the purpose of nitrification (Oladimeji, Olufeagba, et al., 2020; Zou et al., 2016). However, clogging and insufficient oxygen levels in the grow bed are common problems observed during the long‐term operation of the media‐filled‐type system.

Modern aquaponic structures are designed for any size ranging from tabletop, and village size to large commercial sizes as against traditional models which were bogus and fixed (Endut et al., 2010). This speaks of the flexibility and functionality of the aquaponics system. The size of the structure is rightly associated with the quantity of fish grown. Since for each 0.5 kg of fish, 6.8–11.3 kg of vegetables can be produced; the size would usually be determined by funds available and intended use (Nelson & Pade, 2017). In a larger aquaponic structure, the resource costs are higher, although more income may be realized. A study in Jamaica established that a single‐unit system (i.e., a fish tank to two vegetable tanks) is adequate for food security but insufficient for income realization (Pade and Nelson, 2005; Jamaica Fun Farm, 2010).

## 3 ADVANTAGES OF AQUAPONICS PRODUCTION SYSTEM

### 3.1 Water conservation

Large‐scale production of food fish is often done in cramped or limited spaces for various reasons ranging from ease of management to maximal use of limited resources for maximal output. However, these living conditions have their implications, one of which is ridding the water of ammonia that is excreted and highly toxic to the fish (Ogah, Kamarudin, Nurul‐Amin, & Edaroyati, 2020). To mitigate this, water used for commercial‐scale fish farming is periodically changed to avoid damaging and detrimental effects on the fish reared and by extension, the yield obtained (Oladimeji, Olufeagba, et al., 2020). Aside from the continuous wastage of scarce resources in the management of static aquaculture systems, the water renewal protocol is highly inefficient to rid the system of waste. The ammonia is further transformed into nitrate by the action of bacteria, thereby deteriorating water quality and affecting the fish's well‐being at high concentrations (Love, Fry, et al., 2015; Love, Uhl, & Genello, 2015; Pulvenis, 2016). Fortunately, despite this deleterious outcome, nitrate is a crucial nutrient needed by the plant for growth. Hence, the corearing of plants with such large‐scale fish production as seen in the aquaponics production system helps to absolve the nitrate in the water. Therefore, this brings the concept of water reuse and conservation rather than the need to constantly replace it with fresh water. Some authors have reported 90% less water usage through aquaponics compared to conventional commercial fish and crop production systems (Love et al., 2014).

There is no doubt that the aquaponics production of food is a more sustainable venture and efficient water conservation technique adaptable for both developed and developing countries of the world. The principle of recirculation and water reuse with high efficiency is made possible by the integration of the fish and the hydroponics plants in a stable aquatic environment (Oladimeji, Okomoda, et al., 2020). As the hydroponics portion of the system can recover dissolved nutrients from the system, the waste available in the circulating water is substantially reduced, therefore less water is discharged. The only need for freshwater in the system is to account for losses occasioned by plant transpiration, evaporation from the water surface, and flushing of settled solid wastes from the system. The resultant effect of this is that the system requires approximately lower water (about 2%) compared to what is conventionally needed for irrigated farms for the production of a similar number of plants (Rakocy et al., 2004). Therefore, aquaponic production allows efficient production of food commodities in areas where resources for production such as water and arable land are limited or have highly competitive uses.

### 3.2 Improved growth rate and yield

Currently, there is an increased burden to improve conventional farming practices to meet the geometric demands of the ever‐growing human population. Consequently, the pressure is translated to obtaining more farming inputs such as chemical fertilizers, herbicides, pesticides, and fungicides (Love et al., 2014). Without these high costing farm inputs, conventional farming productivity and profitability reduce due to reduced soil fertility. Unlike the conventional farming system, the aquaponics production system is hinged on sustainable practices of nitrogen cycling and continuous watering of the plants with nutrient‐rich wastewater (i.e., nitrate produced by fish and consumed by plants). As a result, the plant and also fish grow faster with less input or external influence (Love, Fry, et al., 2015). It is clear then that the adoption and commercialization of a sustainable food production system such as aquaponics could improve food production within a relatively short period and with a higher standard than what is currently been achieved with a conventional system. This was demonstrated recently by Oladimeji, Okomoda, et al. (2020) who reported that pumpkin production and yield in the aquaponics system were about 5‐ and 11‐fold higher in performance when compared to irrigated land and nonirrigated land, respectively. The authors also found that fish yield was 29% and 75% higher using the aquaponics system compared to the recirculatory and static aquaculture systems, respectively.

### 3.3 Food security and space efficiency

Aquaponics is proposed as a reliable source of food production, as it almost always has a guaranteed success rate in food production except in cases of system breakdown (Rakocy, 2012). This success may be based on the fact that the growing conditions (light, water parameters, and flow rates) can be controlled, so the fish and plants are never exposed to extreme frost, heat, rain, or other bad weather conditions. This makes aquaponics a viable alternative for countries/areas that are plagued with unfavorable weather conditions (i.e., countries with extreme temperatures and drought). Another positive area of aquaponics is that it can be set up almost anywhere to grow fish and crops, provided there is electricity and water. Innovators have come up with the use of solar panels, and wind turbines for power and rain collectors for the water supply (Love, Uhl, & Genello, 2015). Another edge of an aquaponic system for growing crops is the fact that it efficiently utilizes space. This means that contrary to the needed horizontal open spaces used in the conventional system, farmers can rather work in smaller spaces with grow beds designed for vertical orientation. The consequence of this is that commercial‐scale production of food commodities can be done in relatively smaller spaces (Al‐Hafedh et al., 2008).

### 3.4 Less manual labor

Aquaponics can be argued to have lower maintenance as regards labor as weeding is no longer required for the crops. Hence, after the initial intensive cost of labor to set up the system, it runs by itself and requires about half an hour of monitoring or human intervention to keep the system going (Liang & Chien, 2013). The bulk of this intervention has to do with feeding the fish and monitoring the water quality parameters. Other aspects like inspecting the plants for insect infestation and diseases only require occasional attention (Rakocy, et al., 2006a).

### 3.5 Pollution reduction and energy consumption

Another advantage of aquaponics production system over traditional conventional food systems is its environmental‐friendly nature. This is because no heavy earth‐moving machines or equipment are needed to till the soil; hence, the destruction of soil structure and the attendant pollution is eliminated in one swoop using the aquaponics system (Oladimeji, Okomoda, et al., 2020). Skygreen farms have patented what is referred to as “The world's first low carbon hydraulic commercial farming system.” This is a vertical farming system that rotates the plant beds around different positions for optimum sunlight powered by a water hydraulic system as opposed to burning fossil fuel (Nandy, 2020). These researchers claim that the conventional power required to light up one bulb (i.e., 40 W) is the equivalent of electricity needed to power one 9‐m‐tall tower of the system. This they said can sufficiently produce 1 ton of leafy green vegetables every other day (Nandy, 2020).

### 3.6 Organic pest control and economic saving

Most aquaponic systems are maintained indoors (glasshouses, greenhouses, screen‐house, etc.) or under a shade. These physical barriers reduce insect pest incidence and facilitate organic intervention when required (Resh, 2008). This affords the plant the best opportunity for growth and development, hence consequently higher output and yield. Also, the cut in the production costs associated with the conventional agricultural system such as the purchase of fertilizers, fuel for tilling machinery, weeding and other traditional agricultural practice makes the aquaponics system very attractive and cost‐effective/saving. Reports by Love et al. (2014) indicate that 30–75% of survey respondents made more profits between the first to third years of farming using the system compared to conventional agriculture systems.

### 3.7 Adaptability to urban areas and possible solution to farmer–herdsmen clashes.

Today's society faces major challenges due to the continuous movement of individuals from rural to urban areas with motives ranging from job hunting, academic pursuits, and general convenience. This has created an artificial paucity of rural farmers thereby limiting agricultural production. Urban aquaponics could be one of the ways to regain balance as food can be produced even in urban areas. This is being practiced widely in many cities of the world such as Islamabad (Sheikh, 2006), Milwaukee and Melbourne (Laidlaw & Magee, 2014), Kuala Lumpur (Man et al., 2017), and Singapore (Nandy, 2020). Conventional agricultural farming in most developing countries (especially in Africa) sometimes results in conflict between the crop farmers and the cattle or nomadic herdsmen. This resource control problem over grazing routes and water availability is the source of the deadly age‐long violent in many parts of Africa with scores of mortality recorded over time (Ajuwon, 2004; Fasona & Omojola, 2005; Udo et al., 2019). Aquaponics as a means of fish and crop production in an enclosed environment presents a unique opportunity for solving this resource‐use conflict in most parts of Africa. This could be considered a fundamental Government policy not only to boost farmers' income (through production diversification of crops and fish) but protect life and properties.

## 4 CHALLENGES IN AQUAPONICS

Despite the benefits of the aquaponics system, it is not without its share of challenges. Thus, it may not live up to its full potential of providing food security and environmental conservation if some things are not in place. The growth impediments range from obvious issues of high‐power needs, and start‐up capital, to obscurities like the effect of its commercialization on existing production/marketing structures and public perception.

### 4.1 Start‐up capital

A major challenge lies in start‐up capital. The survey by Love, Fry, et al. (2015) found that a minimum of 1000 m^−2^ is required for farmers to break even in the commercial utilization of the aquaponic system in the first year. Respondents in this survey admitted having invested about $5000–$9999 in start‐up and the median quantity of fish and plants harvested ranged between 23–45 kg/year and 45–226 kg/year, respectively. More so, there was no correlation between the amount of money invested and self‐reported profitability. This paints a dull picture of aquaponic investments and especially so for growers in developing nations (who might need to rely on this farming system as a means of livelihood and or income). However, one way to mitigate the state‐up cost will be to use cheap locally available material for the construction of the aquaponics production system (Oladimeji, Olufeagba, et al., 2020). This could also include research into recycling of plastic waste as a component of the system thereby turning waste into wealth. It is important to state that improvisations on the structural set‐up of the aquaponics production system in terms of plant troughs, media beds, filters, sludge collectors, and substrates are not uncommon in previous research (Love et al., 2014; Oladimeji, Okomoda, et al., 2020).

### 4.2 Power for operating the system

Another area that challenges the productivity and profitability of aquaponics is power (for water pumps, aeration, sensors, and lightning among others). Aquaponics is put forward as a way of reducing hunger, malnutrition, as well as providing an additional income stream (Tyson et al., 2011). However, in third‐world countries, the power supply is unstable and epileptic. Aside from the few users of solar and wind energy (serving as supplements to fossil fuels), there has not been any recorded successful commercial use of other alternative power sources; thus, it has remained a challenge. In a survey carried out by Laidlaw and Magee (2014), two community‐driven aquaponic farms, Sweet Water Organics (SWO), Milwaukee, and Centre for Education and Research in Environmental Strategies (CERES) aquaponics, Melbourne, were unable to continue production on a viable scale within 5 years of operation. This was chiefly due to power cost among other factors. In most developing countries, therefore, the adaptability of this technology on a large scale may be more challenging since there is an epileptic power supply. Therefore, more research is needed to determine the usability and efficacy of intermitted recirculation time on the production characteristics of fish and crops in an aquaponics production system. This could help cut down the cost of power used for production.

### 4.3 Nutrient availability in the aquaponic system

Challenges have remained obstinate in the areas of nutrient availability with certain required nutrients by plants poorly supplied to the fish through the feed. This is understandable as the fish do not require these nutrients in the same quantities as the plants, thus it has to be supplemented in the system (Eck et al., 2019). Hobbyists have stuck to “improvised homemade supplements” for many of the nutrients. Some have used ash of banana peel to supplement potassium and sea salt to supplement magnesium, zinc, and iron (depending on where it is sourced). The use of these materials has solved the problems to various degrees but there has not been adequate scientific documentation on the preparation, use, and effectiveness of such supplementations (Jones, 2002). The use of chelates and other synthetic supplements is still sketchy (Love, Fry, et al., 2015). However, since the requirement of the plant also differs, research on the optimization of the performance of different herbs/crops in the aquaponics production system without a need for supplementation is needed (Ogah, Kamarudin, Nurul Amin, & Puteri Edaroyati, 2020). This is not just important for scientific documentation but could constitute a low‐cost recommendation to new adopters of aquaponic technology in rural communities.

### 4.4 Balancing plant–fish ratio for effective production

Optimizing the plant‐to‐fish ratios has been an issue of long debate among researchers too (Buzby & Lin, 2014; Oladimeji, Olufeagba, et al., 2020; Rakocy et al., 2016; Tyson et al., 2011). Lennard and Ward (2019) in their study compared three methods for this purpose. The first method was by Rakocy (1999) who attempted to optimize nutrient production and utilization by increasing the feed fed to the fish. The second method was that of Lennard and Ward (2019) who developed a new and unique method of determining aquaponic feeding rate ratios. The outcome of this approach was a complex mathematical model that was used to predict the amount of fish feed required to grow a known number of lettuce plants. The third approach used by Stuart et al. (2016) was the nitrate determination method. The author ascertained from the study that the method does have some validity as a hypothesis; however, scientific testing by the UVI raises questions about its validity as a design methodology. More research is needed in this regard to set a workable ratio for the economic viability of different plant and fish combinations in an aquaponics production system. Although some research have be done to improve production and cost efficiency has been attempted in various aspects of the aquaponics system, there seems to be a wide gap between the sustainability promise and the current state of things if some cost‐effective actions are not taken. Much of this research would be interdisciplinary and hence require collaborations with many other fields.

### 4.5 Treatment of diseases and pests in the system

Another major challenge is the growing of crops with minimal use of “safe” pesticides. Despite the perceived advantage of lower incidence of pest or disease attacks on indoor‐grown plants in aquaponic systems, some authors have reported having to tackle aphids regardless (Wilson, 2005). Although they might be less susceptible to attack from soil‐borne pests and diseases, Wilson's (2005) study shows that indoor‐grown plants may be subject to many of the pests and diseases that afflict field crops. Finding a solution to this will require developing more disease‐resistant plant varieties, discovering a wider range of beneficial insects and other biological control agents, and developing management protocols to control pest and disease problems in the future (Okemwa, 2015). The same can be said with the treatment of diseased fish in the recirculatory component of the aquaponics system. The rapidity of the spread of disease in a closed system like aquaponics system could lead to the infection of all fish tanks. Aside from the fear of the persistence of these pathogens in the system long after the batch of fish has been discarded, the use of antibiotics could lead to the development of resistant variants in the system. It is therefore important to disinfect the whole system after every batch of production to prevent the spread of disease to new production cycles. Also, the use of biodegradable treatment methods such as plants with bioactive components could help control diseases and prevent the development of resistant variate (Anupa et al., 2021).

## 5 CONCLUSION

The aquaponics production system is no doubt an eco‐friendly option for 21st century food production that integrates the soilless production of plants (hydroponics) with a recirculatory aquaculture system. Despite years of development of this system, there are still many research areas to be exploited to provide information that can help simultaneously improve the production of plants and fish. While a lot of these research areas have been suggested during this review, it is important to mention that the identification of allied aquaponics technologies is an important strategy for improving the functionality of the system. This may involve the integration of the aquaponics system with other food production systems which may result in increased efficiency/productivity, reduction in waste disposal, as well as reduced energy and water usage. While we have expressed the potential of the aquaponics production system as a panacea to solve several problems in Africa and ensure food security, it is, however, sad that the modalities for domesticating this technology are lacking. Therefore, the extra cost of the solar plants may be incurred in the construction and running of the aquaponics system in Africa. The sustainability of this kind of setup and the profitability of the venture could also be the focus of research in the future.

## FUNDING INFORMATION

A grant from the Malaysian Government under the Higher Institution Centre of Excellence awarded to the Institute of Tropical Aquaculture, Universiti Malaysia Terengganu.

## CONFLICTS OF INTEREST

There is no conflict of interest for this review.

## ETHICAL APPROVAL

Not applicable.

## CONSENT TO PARTICIPATE

Not applicable.

## Data Availability

Data for this research were online publications from the Web of Science as contained in the references in this manuscript.
